# The Influence of Socioeconomic Characteristics on Anthropometry, Nutrition Knowledge, and Attitude of People Living With HIV/AIDS Attending Special Treatment Center (STC) National Hospital Abuja, Nigeria

**DOI:** 10.3389/fnut.2021.737381

**Published:** 2022-01-21

**Authors:** Ijioma Okorie, Adaeze Christiana Okorie

**Affiliations:** ^1^Department of Human Nutrition and Dietetics, Michael Okpara University of Agriculture Umudike, Umudike, Nigeria; ^2^Dietetics Unit, National Hospital Abuja, Abuja, Nigeria

**Keywords:** PLWHA, nutrition knowledge/attitude, body mass index/skinfold thickness, socioeconomic characteristics, nutrition assessment

## Abstract

The work is on the influence of socioeconomic status of people living with HIV/AIDS (PLWHA) on their anthropometry, nutrition knowledge, and attitude, attending special treatment center (STC) in National Hospital Abuja, Nigeria. A cross-sectional study design was carried out in the STC and a total of two hundred and seventy [270 (150 men and 120 women)] PLWHA were used for the study. The PLWHA attend STC once in 2 months and subjects were grouped into 16 giving 4 groups per week since the center runs Monday through Thursday weekly and by 2 months, there were altogether 32 groups. Socioeconomic characteristics, nutrition knowledge and attitude, and anthropometric data were collected. Data were analyzed using descriptive statistics, chi-square, and regression analysis. The study revealed that 41.1% of PLWHA had poor knowledge of nutrition, 29.1% had both fair and good knowledge while 0.7% had excellent knowledge. A total of 52.8% had a positive attitude toward nutrition. The skinfold status of the respondents showed that 56.0% were malnourished, while their body mass index (BMI) revealed that 49.3% were normal. Gender (being female) and age (being young) had a significant (*P* < 0.01) influence on the BMI of PLWHA, while being female significantly influenced their skinfold status. Self-employed and being female were strongest (*P* < 0.01) positive predictors of nutrition knowledge, while low education had a negative influence on their attitude toward nutrition. Good nutrition knowledge, attitude, and improved socioeconomic characteristics are important tools in the care process of PLWHA, for sustainable healthy PLWHA, leading to a healthy society at large.

## Introduction

Bijlsma (1) reported that AIDS is a disease caused by a retrovirus known as HIV which attacks and impairs the natural defense system of the body against disease and infection. Bijlsma (1) further stressed that HIV is a slow-acting virus that may take years to produce illness in a person. During this period, the defense system of an HIV-infected person is impaired, and other viruses, bacteria, and parasites take advantage of this “opportunity” to further weaken the body and cause various illnesses, such as pneumonia, tuberculosis, and oral thrush.

Human immunodeficiency virus (HIV) is one of the worst infections that have decimated the human population, especially in resource-compromised societies such as developing African countries. In Africa alone, nearly 25 million are living with HIV/AIDS, the vast majority of the adults in the prime of their working and productive age (2–4). About 15 million people in Africa are reported to have died of AIDS, while about 12.1 million children have been orphaned in Africa because of the infection (2). HIV infection constitutes a global public health emergency and is most prevalent in areas of the world where undernutrition is a serious concern. Despite tremendous advances in care for HIV infection and increased funding for treatment, morbidity and mortality due to HIV/AIDS in developing countries remain unacceptably high.

Globally, the HIV burden is least in the richest countries, however, in sub-Saharan Africa countries HIV prevalence is highest in the wealthiest countries. Nigeria is reported to be the second in the world with the highest number of new HIV infections reported each year and an estimated 3.7% (about 3.4 million) people of the population are estimated to be living with HIV (5). However, the rate of infection has reduced in Nigeria, as reported by Nigeria National HIV/AIDS Indicator and Impact Survey (2019) that, the prevalence of HIV in Nigeria is 1.4%.

Despite the recent reduction in the rate of infection, there is a need to scale up nutrition assessment and education is thus pertinent to slow the progression of the disease (4, 6). Nutrition appears to be one of the major factors that play a critical role in the prevalence of HIV/AIDS, yet it is taken for granted (4). Nutrition for rich people is satisfaction in the fact that there are no food items that are beyond reach; for the poor, there is consolation in the fact that only what can be afforded is eaten and for the average majority there is solace in the fact that there is no obvious problem with what to eat (4, 7). Knowledge means the ability to pursue and use information, while attitude indicates the result of making a reaction through some ways in some situations. Therefore, nutrition is a significant determinant of individual anthropometric status, which in deficiency state results in malnutrition. However, having access to food items is not enough but knowledge of nutrition and attitude toward nutrition is paramount to improve the nutrition and health status of people living with HIV/AIDS. Thus, the present study is targeted at the influence of socioeconomic status of people living with HIV/AIDS (PLWHA) on their anthropometry, nutrition knowledge, and attitude, attending a special treatment center (STC) in National Hospital Abuja, Nigeria.

## Methodology

### Study Design

The study was conducted at the STC, an out-patient clinic in National Hospital Abuja, Nigeria. National Hospital Abuja is a multidisciplinary tertiary hospital located in the Central district (phase II Garki) of Federal Capital Territory, Abuja. A cross-sectional study design was used to assess the influence of socioeconomic status on anthropometry, nutrition knowledge, and attitude of people living with HIV/AIDS attending STC Abuja, Nigeria. The population of the study was people living with HIV/AIDS attending STC in National Hospital Abuja, who are aged 18–64 years old.

#### Choice of Respondents

The respondents were out-patient HIV-affected individuals, and this was because the study has no required capacity to assess in-patient HIV-affected individuals. The study also considered those that have been HIV-positive for 2 years and above, in order to have a clear understanding of how socioeconomic status could influence their attitude toward nutrition and its knowledge and anthropometry, so as to draw a better inference in the study.

### Sample Size Determination and Sampling Procedure

The sample size was calculated using the formula for a known population by Yamane (8):

*n* = *N*/1 + *N*(*e*)^2^
*n* = sample size
*N* = population size
*e* = level of precision required for the results
*N* = 5,405; *e* = 0.05.

$$\begin{array}{l} \frac{5,\text{​}405}{1+5,\text{​}405{\left(0.05\right)}^{2}}=360:\text{Thus},\text{ 10}\%\text{ dropout was};\;\\ 36+360=396. \end{array}$$

Therefore, the sample size for the study was three hundred and ninety-six (396).

Sampling procedure: Proportion allocation was used to select the number of men and women that were included in the study. Thus, the formula: *n*_*i*_ = *N*_*i*_/*N* × *n* Rajiv (9).

*N* = total population, *N*_*i*_ = total number of male/female living with HIV/AIDS, *n*_*i*_ = number of male/female in the *i*th group. *N* = 5,405; *n* = 396, *N*_*i*_ = 2,800 (male)/2,605 (female). However, using the formula above;

For the male; *n*_*i*_ = 2,800/5,405 × 396 = 205.

For the female; *n*_*i*_ = 2,605/5,405 × 396 = 191.

The study population attends STC once in 2 months, and there ~4 weeks in a month. They were grouped since the center runs Monday to Thursday weekly.

For the first 4 weeks, there were 16 groups (groups I, II…IV per week) and by 2 months, the groups were altogether 32 groups. The number of PLWHA [males (205) and females (191)] were spread across the 32 groups, giving 6 male participants (205/32 = 6) and 6 female participants (191/32 = 6). The sample size (396) was the basis for the study, however, the total number of the PLWHA (both male (150) and female (120) subjects) that were used for the study was two hundred and seventy (270), which was about 68.2% response rate. Thus, in each group, there was a total of 12 subjects both male and female, but this was reduced to nine subjects (5 male and 4 female) per group due to the response rate. In each of the groups, the subjects were segregated by sex, and then, simple random sampling by balloting without replacement (Yes/No) was used to select the number of subjects required for the study on each day of visit. Everybody was given an equal opportunity to be picked and those that picked “Yes” were used for the study.

Ethical approval was obtained from the Ethical Committee National Hospital Abuja, allowing the researcher to use their subjects for the study.

Trained clinical nutritionists, registered dietitians, nurses, and laboratory scientists were used in the study to evaluate the out-patient HIV-affected individuals, through one-on-one interviews. Before administration of the questionnaires, written and/or oral informed consent was obtained from each patient after being assured of the confidentiality of volunteered information. Nutrition education including dietary counseling (given by the researcher) was the major incentive given to the participants after the data collection.

### Data Collection

The structured and validated questionnaire was used to collect information on the background information and socioeconomic characteristics of PLWHA, their nutrition knowledge, and their attitude toward nutrition. The questionnaire was administered to the respondents during their scheduled appointments in the STC National Hospital, Abuja.

### Anthropometric Measurement

Weighing scales and meter rules were used to obtain weights (in kilograms) and heights (in meters) of subjects, respectively, using standard procedure: subjects were weighed three times with minimal clothing and the average of the reading was recorded to the nearest 0.1 kg. The height was measured with subjects standing against a wall, their heel, buttocks, and vortex against the wall for their heights to be measured in centimeters. An average of two readings was recorded to the nearest 0.1 cm. Their body mass index (BMI) was calculated using (weight in kilograms/height in meters^2^) and recorded. World Health Organization (WHO) (10) classification of BMI (underweight, overweight, and obese) was used to classify the subjects into underweight, overweight, and obese. Skinfold thickness was also measured and calculated to the nearest 0.1 cm using a skinfold caliper.

### Determination of Knowledge and Attitudinal Questions

Knowledge was assessed by the use of knowledge questions where a grading method was used to develop a nutritional knowledge index. A list of 15 nutrition-related questions was presented to the respondents and responses were marked and scored. For every correct response, 1 mark was given while for each wrong response a score of 0 was given. A respondent can score a maximum of 15 and a minimum of 0 in the knowledge section.

The attitude was assessed using a three-point scale (Likert scale). This method was used for grading the intensity of attitudes of the respondent toward adequate nutrition. The Likert scale comprised of one positive, a middle option that captures attitudes that are still uncertain and one negative, a score of 1 was given to “disagree,” a score of 2 was given to “do not know,” and a score of 3 was given to “agree,” so that a respondent could score a maximum of 45 and a minimum of 15 in the attitude section.

### Data Analysis

Data were collected on the socioeconomic characteristics of the respondents, their nutrition knowledge, and attitude toward nutrition using a structured questionnaire, and analyzed. Nutritional status using anthropometric measurements (weight, height, and skinfold thickness) were measured and analyzed. The BMI was calculated and compared to WHO BMI standard, underweight (<18.50 kg/m^2^), normal (18.5–24.99 kg/m^2^), overweight (25–29.99 kg/m^2^), and obese (>30 kg/m^2^), and nutrition knowledge score was graded thus, Poor (39%), Fair (40%−49%), Good (50%−69%), and Excellent (>70%) while attitude was assigned thus, Disagree (0–1.50), Undecided (1.51–2.25), and Positive (2.26–3.00).

### Statistical Analysis

Descriptive statistics (frequency and percentages) were used to analyze the categorical and continuous variables such as socioeconomic characteristics, knowledge, and attitude grades of the respondents. Chi-square was used to analyze the anthropometric status, while regression analysis was used to determine the influence of socioeconomic predictors on anthropometric status, nutrition knowledge, and attitude of PLWHA.

## Results and Discussion

Table 1 shows the background information and socioeconomic characteristics of the respondents. Few (31.3 and 36.8%, respectively) male and female respondents were aged 34–41 years of old. The higher percentage of this age range living with HIV was because at this age (20–58 years) young people experiment with sex and take risks. It is therefore not surprising that the higher number of the respondents belonged to this age group. The higher number of the respondents who belonged to this age range concurred with previous studies inside and outside of Nigeria (11, 12).

**Table 1:** Background information and socioeconomic characteristics of the respondents.

**Parameters**	**Male**	**Female**	**Total**
	**F (%)**	**F (%)**	**F (%)**
Age
18–25 yrs	11 (7.3)	10 (8.5)	21 (7.9)
26–33 yrs	30 (20.0)	30 (25.6)	60 (22.5)
34–41 yrs	47 (31.3)	43 (36.8)	90 (33.7)
42–49 yrs	33 (22.0)	19 (16.2)	52 (19.5)
50–57 yrs	16 (10.7)	10 (8.5)	26 (9.7)
58–65 yrs	13 (8.7)	5 (4.3)	18 (6.7)
Total	150 (100.0)	117 (100.0)	267 (100.0)
Marital status
Single	25 (16.7)	30 (25.0)	55 (20.4)
Married	101 (67.3)	63 (52.5)	164 (60.7)
Divorced	6 (4.0)	8 (6.7)	14 (5.2)
Widowed	10 (6.7)	17 (14.2)	27 (10.0)
Widower	5 (3.3)	1 (0.8)	6 (2.2)
Separated	3 (2.0)	1 (0.8)	4 (1.5)
Total	150 (100.0)	120 (100.0)	270 (100.0)
Educational status
No formal education	15 (10.1)	14 (11.9)	29 (10.9)
Primary education	14 (9.4)	7 (5.9)	21 (7.9)
Secondary education	57 (38.3)	42 (35.6)	99 (37.1)
Tertiary education	63 (42.3)	55 (46.6)	118 (44.2)
Total	149 (100.0)	118 (100.0)	267 (100.0)
Occupation
Civil servant	62 (41.9)	38 (37.6)	100 (40.2)
Trader	48 (32.4)	45 (44.6)	93 (37.3)
Farmer	9 (6.1)	11 (10.9)	20 (8.0)
Artisan	13 (8.8)	4 (4.0)	17 (6.8)
Pensioner	11 (7.4)	2 (2.0)	13 (5.2)
Student	3 (2.0)	1 (1.0)	4 (1.6)
C. Driver	2 (1.4)	0 (0.0)	2 (0.8)
Total	148 (100.0)	101 (100.0)	249 (100.0)
Average monthly income
< N = = 10,000	36 (25.4)	50 (47.6)	86 (34.8)
₦ 10,000–20,000	18 (12.7)	9 (8.6)	27 (10.9)
₦ 21,000–30,000	19 (13.4)	9 (8.6)	28 (11.3)
₦ 31,000–40,000	21 (14.8)	12 (11.4)	33 (13.4)
₦ 41,000 and above	48 (33.8)	25 (23.8)	73 (29.6)
Total	142 (100.0)	105 (100.0)	247 (100.0)

*F, frequency; %: percentage; C. Driver, commercial driver*.

Most (67.3%) of the male respondents were married while more than half (52.5%) of their female counterparts were also married. This suggests that men were more infected than women by HIV. This finding does not support the report by UNAIDS (13) who reported that more women were living with HIV/AIDS in sub-Saharan African than HIV/AIDS positive men. This study concurred with the study done in Nigeria (14).

The educational status of the respondents revealed that some (42.3 and 46.6%) of both male and female respondents, respectively, had tertiary education while few (38.3%) and (35.6%) respectively of both male and female respondents had secondary education. Therefore, amongst all the respondents, few (37.1%) and some (44.2%) had secondary and tertiary education respectively, while few (10.9%) of them had no formal education.

This finding is similar to previous studies from Ezechi (15) and Aliyu et al. (16) who stated that the high educational attainment of the respondents could be attributed to the location of the study area. Some (41.9%) of the male and few (37.6%) of the female respondents were civil servants, while few (32.4%) of the male and some (44.6%) of the female respondents were traders. The highest average monthly income amongst the male respondents was ₦41,000 and above (33.8%) while amongst the female respondents some (47.6%) earned < ₦10,000 monthly. The result also revealed that amongst the total respondents, a few (34.8%) of them earned < ₦10,000 monthly. Although the majority (93.1%) of the respondents were engaged in income-generating activity, their incomes of ₦10, 000 per month, or less indicated that the respondents were employed in low-paying jobs. This finding is in agreement with the study done by Sakhile et al. (17), where the majority of the respondents earned less income because they were engaged in low-paying jobs.

Table 2a reveals the nutrition knowledge status of the respondents, thus, less than half (41.1%) of the respondents had poor knowledge of nutrition, while 29.1% had both fair and good knowledge of nutrition, however, very few (0.7%) had excellent knowledge of nutrition. The respondents' mean nutrition knowledge score was 38.63 ± 17.53. The study revealed that more respondents had poor knowledge on adequate nutrition and this could be due to insufficient nutrition education during their visit to the hospital and/or due to being absent-minded and probably depressed as a result of their present condition. However, the importance of adequate nutrition for PLWHA cannot be overlooked. Akumiah et al. (18) reported that nutrition knowledge score was greater in those (PLWHA) that received sufficient education on nutrition compared to those who did not receive sufficient nutrition education. Furthermore, poor nutrition knowledge plays a key role in the rapid progression of HIV, and this is in line with the study by Muthamia et al. (19) who reported that adequate nutrition knowledge is also among key factors that determine the quality of life among PLWHA, although they have been largely overlooked, especially in resource-limited settings.

**Table 2a:** Nutrition knowledge status of the respondents.

**Nutrition knowledge status**	**Frequency**	**Percentage**
Poor (<39%)	116	41.1
Fair (40–49%)	82	29.1
Good (50–69%)	82	29.1
Excellent (>70%)	2	0.7
Total	282	100.0
**Mean and SD of nutrition knowledge questions**		
**Parameters**	**Frequency**	**Mean** **±SD**
Definition of adequate nutrition	282	1.3262 ± 0.94697
Most important meal of the day	282	1.0638 ± 0.99973
Main function of starchy foods	282	0.9716 ± 1.00137
Good source of carbohydrate	282	1.0851 ± 0.99814
Function of protein rich foods	282	1.0780 ± 0.99872
Example of protein rich food	282	1.3121 ± 0.95175
Example of complete protein	282	0.3617 ± 0.77116
Function of dairy products	282	0.7589 ± 0.97222
Major nutrient in dairy product	282	0.7872 ± 0.97884
Main function of vitamin	282	0.4113 ± 0.80982
Orange is rich in vitamin C	282	0.7730 ± 0.97564
Vitamin A helps in	282	0.0426 ± 0.28912
Minerals are supplied to the body by consumption	282	1.4823 ± 0.87758
No. of glasses of water that should be taken daily	282	0.1348 ± 0.50223
Nutrition knowledge score (%)	282	38.6288 ± 17.52621

Table 2b shows the attitude of respondents toward nutrition. A little more than half (52.8%) of the respondents had a positive attitude toward nutrition while 47.2% of them were undecided about their attitude toward nutrition. The mean attitudinal score of the respondents on nutrition was 2.36 ± 0.36. The study showed that more of the respondents had a positive attitude toward nutrition. This validates the choice of respondents concerning the duration of the disease, which supports the view that prolonged exposure to HIV influences the endocrine secretion levels which in turn could influence their attitudes toward adequate nutrition, and thus, may indirectly affect their nutritional status. Furthermore, it could also be due to the fact that eating a variety of food in moderation is key to adequate nutrition and also the respondents had a good attitude that maintaining a healthy diet is their responsibility and that skipping meals is not good for their health. This is in agreement with the studies done by Bukusuba et al. (20) and Deyika and Thahira (21) that a good number of PLWHA understood that consumption of a balanced diet, fruits, and vegetables is necessary for good health, and skipping meals would increase the side effect of antiretroviral treatment. This, therefore, suggests that nutrition knowledge alone could not be sufficient enough to change dietary habits and/or practices, however, a positive attitude toward nutrition is important to healthy eating and more so, accessibility to nutrition sources.

**Table 2b:** Nutrition attitude status of the respondents.

**Attitude status**	**Frequency**	**Percentage**
Undecided (1.51–2.25)	133	47.2
Positive (2.26–3.00)	149	52.8
Total	282	100.0
**Mean and SD of attitude questions**		
**Parameters**	**Frequency**	**Mean** **±SD**
Preparing a balance meal is time consuming	282	2.3830 ± 0.80177
It is important for PLWHA to know about preparing an adequate meal	282	2.3121 ± 0.72705
It is not vital to eat an adequate meal if already on antiretroviral (ART)	282	2.2518 ± 0.82039
A nutritious meal can come from one's own small garden	282	2.3191 ± 0.82935
1 should eat fruits when I feel like	282	2.1170 ± 0.88309
Vegetables must be over-cooked to kill microbes	282	2.2801 ± 0.81984
Self-view of body weight is important	282	2.3865 ±0.73758
Hygiene is as important as an adequate meal	282	2.4858 ± 0.72191
Taking supplements is better than natural foods	282	2.4149 ± 0.77885
Processed foods are generally better than natural foods	282	2.2766 ± 0.85709
Eating variety of foods in moderation is a key to adequate nutrition	282	2.4468 ± 0.78169
Maintaining a healthy diet is my responsibility	282	2.5603 ± 0.68402
Skipping meals is not good for my health	282	2.4291 ± 0.79831
Attitude score	282	2.3587 ± 0.36345

Table 3 reveals the anthropometric status of the respondents. Thus, 49.3% of the respondents had normal weight, of which 57.4% were male and 39.2% were female. The female respondents were found to be overweight more than their male counterparts; 25.0% of men and 32.5% of women had BMI equals 25.0–29.9 kg/m^2^. More so, 20.0% of female respondents were obese while 7.4% of their male counterparts were obese. There was a significant difference (*P* < 0.05) in the BMI status between male and female respondents, thus it was discovered that men had lower fat than their female counterparts. This is in line with studies by Walsh et al. (22) and Gideon and Olamide (23) that reported essentially lower fat in men with about 3.0% of the total weight vs. 12.0% in women. It has been reported that the higher fat percentage in women is due to sex-specific fat, such as breast, uterus, and other sex-related fat deposits (23).

The result also showed the skinfold status of the respondents where 56% of the respondents were malnourished; more than sixty-four percent (64.2%) of the men were malnourished than their female counterparts (45.8%) while 54.2% of the female had normal skinfold compared to 35.8% of the men. The chi-square showed that there was a significant difference (*P* < 0.05) among the male and female respondents concerning their skinfold status. Using triceps skinfold status, the male respondents were malnourished compared to the women and the percent body fat was significantly higher and/or normal in women than in men. This could be due to the fact that females tend to lay down more subcutaneous fat layers than males during the growth spurt at puberty. The finding of this study is in line with the study by Gideon and Olamide (23). However, malnutrition is a serious danger for PLWHA, even at the early stages of HIV infection when no symptoms are apparent, HIV makes demands on the nutritional status of the body (22).

**Table 3:** Anthropometric status of the respondents.

**Parameters**	**Male**	**Female**	**Total**	**X^2^ test of sig. (0.05)**
	**F (%)**	**F (%)**	**F (%)**	
Body mass index status (kg/m^2^)				0.002
Underweight	14 (9.5)	7 (5.8)	21 (7.8)	
Normal	85 (57.4)	47 (39.2)	132 (49.3)	
Overweight	37 (25.0)	39 (32.5)	76 (28.4)	
Obese	11 (7.4)	24 (20.0)	35 (13.1)	
Morbid obesity	1 (0.7)	3 (2.5)	4 (1.5)	
Total	148 (100.0)	120 (100.0)	268 (100.0)	
Skinfold status (cm)				0.003
Malnourished	95 (64.2)	55 (45.8)	150 (56.0)	
Normal	53 (35.8)	65 (54.2)	118 (44.0)	
Total	148 (100.0)	120 (100.0)	268 (100.0)	

Table 4 shows the influence of background information and socioeconomic status on the BMI, skinfold, nutrition knowledge, and attitude of the PLWHA. The BMI of the respondents was significantly (*P* < 0.01) positively influenced by gender (being female) but negatively influenced by age (being younger than 34 years). These variables contributed 7.6% in the variability of the BMI of the respondents. Female respondents were observed to have BMI values 2.63 kg/m^2^ higher than their male counterparts while young respondents had BMI values 1.89 kg/m^2^ lower than respondents 34 years and above. Gender was the only socioeconomic characteristic that had a significant (*P* < 0.01) and positive influence on the skinfold thickness of the respondents. The study showed that being female contributed 0.87% to the variability in the skinfold thickness and women had skinfold thickness 5.23 cm higher than the men. Gender (being female) and age (young age) had an influence on the BMI while being female alone influenced the skinfold thickness of the respondents. This could suggest that women are more likely to adhere to treatment regimens and have access to health services than men, and this may have contributed to having a better BMI and skinfold thickness than their male counterparts. Thus, an increase in BMI and skinfold thickness are indications of improvement in physical health which helps to increase self-confidence. However, the study is in contrast with the study by Yen et al. (24) where male patients had a better BMI and skinfold thickness than their female counterparts. They further suggested that better BMI and skinfold thickness contributed to the quality of life of PLWHA, increase self-confidence, infuse a sense of independence wherein they (PLWHA) move around and feel accepted in the environment they stay in.

The result also suggested that young age influenced the BMI of PLWHA. This could be due to the fact that young adults may be particularly susceptible to inadequate dietary intake due to the stigma associated with HIV/AIDS. This may also lead to undernutrition and thus, could have contributed to the low BMI reported in this study compared to those aged 34 years and above. This is in agreement with a study by Lori et al. (25) that young PLWHA is invariably exposed to many of the same risks for higher BMI that are relevant to the general population, however, due to the stigma associated with HIV/AIDS, they tend to live discriminately.

**Table 4:** Influence of socioeconomic characteristics on nutritional status of people living with HIV/AIDS.

**Variables**		**Unstandardized coefficients**		**R square**	***t***	***P*-value**
		**B**	**Std. error**			
	(Constant)	24.247	0.473		51.244	0.0001
BMI	Being female	2.627	0.651		4.034	0.0001
	Being young	−1.886	0.710	0.076	−2.655	0.008
						
	(Constant)	10.951	0.697		15.707	0.0001
Skinfold thickness	Being female	5.232	1.042	0.087	5.022	0.0001

*BMI, body mass index*.

Table 5 reveals that type of occupation and gender were the strongest (*P* < 0.01) and positive predictors of nutrition knowledge of the respondents. The study revealed that being a civil servant, self-employed, or female contributed 11.3% to the variability in the nutrition knowledge scores of the respondents. Respondents who are civil servants had a nutrition knowledge score (14.84%) higher than others, while self-employed respondents had a nutrition knowledge score (10.2%) higher than those that are neither self-employed nor civil servants.

**Table 5:** Influence of socioeconomic characteristics on the nutrition knowledge and attitude of people living with HIV/AIDS.

**Variables**		**Unstandardized coefficients**		***R* square**	***t***	***P*-value**
		**B**	**Std. error**			
	(Constant)	25.903	2.508			
Nutrition Knowledge	Being civil servant	14.843	2.878		10.330	0.0001
	Being self employed	10.232	2.757	0.113	5.158	0.0001
	Being female	6.280	2.004		3.712	0.0001
	(Constant)	2.368	0.033		72.641	0.0001
Attitude	Being female	0.145	0.043		3.373	0.001
	Low EDUCATIONAL Status	−0.151	0.055	0.074	−2.750	0.006
	Being single	−0.118	0.044		−2.667	0.008

Female respondents also had a 6.28% score in nutrition knowledge more than male respondents. From the study, the type of occupation (civil servant and/or self-employed) and gender (female) influenced the nutrition knowledge of the respondents. This could be attributed to their level of exposure and/or quest to living healthy and as such, they may be open to knowledge on nutrition such as dietary diversity and also nutrient-dense foods, as it concerns the management of HIV/AIDS thereby reducing the risk of opportunistic diseases. The study is in line with the study by Muthamia et al. (19) thus, nutrition knowledge influences the choice of nutrient-dense foods that are high in nutrients compared to their (PLWHA) weight.

The attitude of the respondents was significantly (*P* < 0.01) predicted by gender, educational status, and marital status, which contributed 7.4% to the variability of the attitudinal score. The study showed that low educational status and being single negatively influenced the attitude of the respondents toward nutrition. Respondents with low educational status had an attitudinal score of 0.15 points lower than those with high educational status while respondents who are single or not living with their spouse were 0.12 points lower in their attitude compared to those that are living with their spouse. However, female respondents had a higher attitudinal score (0.15 points) than the male respondents. Low educational status and being single had a negative influence on the attitude of the respondents toward nutrition. This may be due to lack of interest which could influence their attitude toward nutrition and/or with low educational status, they may have low-paying jobs with low-income generation and as such may feel less concern toward nutrition with the belief that a good and nutritious diet is expensive. The finding is in agreement to some extent with a study by Liu et al. (26) that most patients with HIV have no education, focus on only the drugs they have been given, and that they have a poor attitude toward nutrition in fulfilling the nutritional needs of the antiretroviral therapy in helping them to live longer with a better quality of life. More so, low and/or no education can predispose PLWHA to inadequate dietary intake which invariably increases the risk of opportunistic diseases, and thus, their immune system is compromised.

## Conclusion

From this study, it can be seen that background information and socioeconomic characteristics such as education and occupation have a significant influence on the anthropometry of people living with HIV/AIDS. Thus, nutrition knowledge and attitude is not a standalone tool, however, proper education, occupation (good paying job), and of course, high income are important variables, which when in place and sustained, will promote the anthropometric and health status of people living with HIV/AIDS thereby leading to a healthy nation at large.

## Data Availability Statement

The original contributions presented in the study are included in the article/supplementary material, further inquiries can be directed to the corresponding author/s.

## Ethics Statement

The studies involving human participants were reviewed and approved by Ethical Committee National Hospital Abuja Nigeria. The patients/participants provided their written informed consent to participate in this study.

## Author Contributions

IO: design of the study, final content developer, data analysis, and data collection, funding, methodology. AO: literature search, data collection, and processing, funding. Both authors contributed to the article and approved the submitted version.

## Conflict of Interest

The authors declare that the research was conducted in the absence of any commercial or financial relationships that could be construed as a potential conflict of interest.

## Publisher's Note

All claims expressed in this article are solely those of the authors and do not necessarily represent those of their affiliated organizations, or those of the publisher, the editors and the reviewers. Any product that may be evaluated in this article, or claim that may be made by its manufacturer, is not guaranteed or endorsed by the publisher.
